# A Case of Cerebral Granuloma and Optic Papillitis due to *Brucella* sp.

**DOI:** 10.1155/2020/5216249

**Published:** 2020-07-17

**Authors:** A. Chiappe-Gonzalez, A Solano-Loza

**Affiliations:** ^1^Hospital Nacional Dos de Mayo, Lima, Peru; ^2^Clínica Angloamericana, San Isidro, Peru

## Abstract

We document a case of a 24-year-old woman who presented with cerebral granuloma and optic papillitis associated to *Brucella* sp. infection, whose diagnosis was made with a brain biopsy and serology tests, with clinical improvement following specific antibiotic therapy. The patient was followed up for over a year without evidence of relapse.

## 1. Introduction

Brucellosis is a common zoonotic infection in many countries, including Mediterranean and Middle Eastern countries. In Peru, the prevalence of brucellosis has been poorly documented, with higher frequency in the cities of Lima, Callao, and Ica probably due to the informal goat farming in these regions.

The transmission to humans is through the consumption of infected unpasteurized animal milk or dairy products, the direct contact with infected animal parts, or the inhalation of infected aerosolized particles. [1].

We present the case of a 24-year-old woman from Lima, Peru, with the diagnosis of a granulomatous occipital lesion secondary to *Brucella* sp. infection that is a very uncommon neurologic manifestation of brucellosis.

## 2. Case Presentation

A 24-year-old woman from Lima, Peru, presented with a nine-month history of persistent right hemicraneal headache preceded by photopsia, associated to nausea and photophobia. Her medical history was relevant for migraine; she worked as a teacher at an orphanage and had travelled within the previous year to Morocco and Spain.

**Figure 1:**
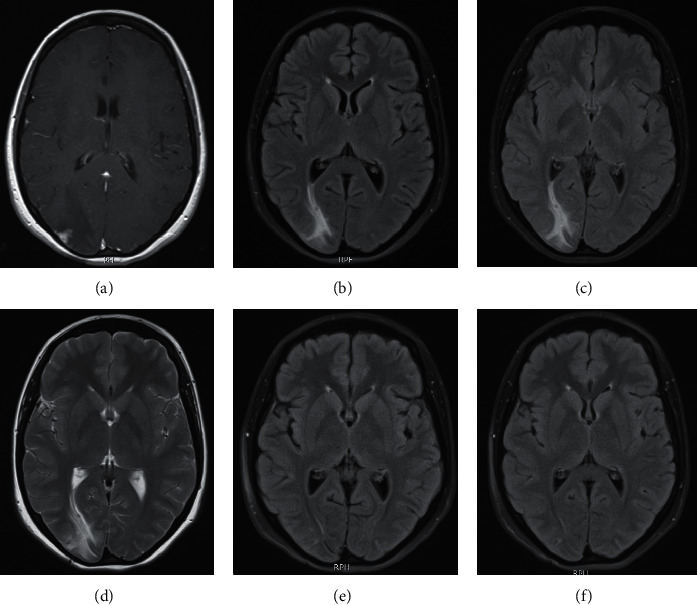
Baseline and evolution of cerebral MRI in neurobrucellosis patient. (a) Initial presentation. (b) 1 month later. (c) 2 months later. (d) 7 months later. (e) 6 months after treatment. (f) 1 year after treatment.

Seven months prior to presentation, she had been evaluated for this complaint; a head computed tomography (CT) angiography was performed which showed a hypodense, right occipital lesion with ill-defined borders and peripheral contrast enhancement (Figure 1); the study was followed by a brain magnetic resonance imaging (MRI), which confirmed the presence of a solid cortical formation of about 0.7 centimeters in the right occipital lobe, with an irregular border, associated with vasogenic edema. A biopsy of the lesion was performed, and it demonstrated a necrotizing granuloma, with no identification of acid-fast bacilli, a negative *Mycobacterium tuberculosis* tissue polymerase chain reaction (PCR), and no evidence of malignancy. The patient was then referred to the Infectious Diseases Department in order to rule out infectious causes of granulomatous brain lesions. Further workup demonstrated negative results for *Histoplasma* antibodies, *Listeria monocytogenes* (IgG), Leptospira (IgG-IgM), Hydatidosis (IgG), *Borrelia burgdorferi* (IgG-IgM), Coxsackie B Ab [1–6], *E*. *histolytica* Ab, *Bartonella henselae* (IgG-IgM), QuantiFERON TB, antinuclear antibodies, ANCA C-P, SS-A antibodies, SS-B antibodies, Rose Bengal, tube agglutination, 2-mercaptoethanol test, prozone phenomenon, and blocking antibodies.

After four months without a definitive diagnosis, she was readmitted after an episode of generalized tonic-clonic seizures. Serum and cerebrospinal fluid (CSF) tests were negative for histoplasmosis, paracoccidioidomycosis, vasculitis, and tuberculosis. Empiric treatment for cerebral tuberculosis (isoniazid 300 mg, rifampicin 600 mg, pyrazinamide 150 mg, and ethambutol 1200 mg/daily plus dexamethasone 6 mg/daily) therapy was started given the high prevalence of tuberculosis in Peru and the finding of a necrotizing granuloma in the brain biopsy, but the headache and photopsia persisted. Evaluation by Ophthalmology, which included fluorescein angiography, found bilateral optic papillitis without signs of uveitis. Further studies for granulomatosis demonstrated a switch in the *Brucella* panel tests, with positive plate agglutinations, positive Rose Bengal test, and positive (1/100) tube agglutination test; these tests were performed in three different laboratories, and all of them reported consistent results.

Specific brucellosis treatment was started with doxycycline 100 mg twice a day, trimethoprim-sulfamethoxazole 160/800 mg twice a day, and intravenous amikacin 1 g daily; she received amikacin only for the first 10 days and the other two antibiotics for twelve weeks. Corticosteroids doses were tapered gradually until total discontinuation at the end of the first month of specific therapy.

Clinical, laboratory, and radiological improvement was seen during the ambulatory 12-month follow-up visit; she did not have any other headache crisis, neither seizures nor visual disturbances.

## 3. Discussion

The clinical spectrum of brucellosis is very heterogeneous, with no specific symptoms that could differentiate it from other diseases; fever and constitutional symptoms are usually present and the physical exam is nonspecific as well. Osteoarticular disease is the most common presentation of brucellosis, but there are other presentations like hepatic, respiratory, cardiovascular, hematologic, or reproductive disorders, with a few rare cases of nervous system disease [1].

In the case presented, seroconversion of the serology results was seen, which supported the diagnosis of *Brucella* sp. infection. Serology still remains the most frequent tool used for the diagnosis of brucellosis. David Bruce developed the tube agglutination test, which measures antibodies against smooth lipopolysaccharide, and it remains nowadays one of the most used laboratory tests for this diagnosis. Titers above 1 : 160 are considered diagnostic in association with a compatible clinical presentation. Other serology tests are based on antibody production against other bacterial antigens [2].

The development of a definitive diagnostic test for brucellosis can be difficult. The absolute diagnosis requires the isolation of the bacteria from blood or tissue samples. The percentage of cases with positive cultures ranges from 15 to 70% [1].

The case we presented describes a focal occipital granuloma and optic neuritis as central nervous compromise. This particularly rare complication of the *Brucella* sp. infection represents 5–7% of total cases and usually has a bad prognosis [3].

Clinical manifestations of neurobrucellosis are heterogeneous and can involve the central and peripheral nervous system. The broad clinical presentations vary from meningitis, encephalitis, polyradiculoneuritis, sensory and motor abnormalities, and cranial nerve affectation to epilepsy, depression, brain abscess, subarachnoid hemorrhage, and even coma. Sensorineural hearing loss is described as an important morbidity [4] Less common neurological manifestations are papilledema, optic neuropathy, radiculopathy, stroke, and intracerebral hemorrhage [5] Papillitis (optic neuritis) has been implicated in the pathophysiology of papilledema; it presents as pain on eye movement, progressing to visual loss and pupillary defect [6] There are other even more rare neurologic manifestations like isolated intracranial hypertension, Guillain-Barre syndrome, solitary extra-axial posterior fossa abscess, cerebral venous thrombosis, and subdural hemorrhage [7, 8].

The most common clinical manifestation of neurobrucellosis is meningitis or meningoencephalitis, representing 50% of total cases; this central nervous compromise may lead to lymphocytic pleocytosis, cranial nerve damage, and intracranial hypertension [9] The CSF of our patient had no relevant alterations, presumably because it was an isolated parenchymal lesion with no CSF infiltration.

The presence of spinal granuloma or abscess secondary to *Brucella* sp. infection may be confused with other chronic infections like tuberculosis and syphilis. Therefore, it is important to rule out other possible entities such as mycobacterial, bacterial, parasitic, and fungal infections, as well as sarcoidosis and vasculitis, among others. In our patient, many of these conditions were excluded by CSF cultures, serum analysis, and brain biopsy [9].

The diagnosis of neurobrucellosis is based on the presence of neurologic manifestations not explained by other neurologic diseases, evidence of systemic brucellar infection and the presence of inflammatory changes in cerebrospinal fluid [10]. Also, radiological compromise can be divided into four types: normal, white matter changes, vascular insult, and inflammatory changes [11].

In addition, treatment of neurobrucellosis is uncertain, but the literature recommends a 3-drug combination based on doxycycline plus rifampin plus aminoglycoside or trimethoprim-sulfamethoxazole, or a third-generation cephalosporin. Duration of treatment is not well established, but experts recommend no less than 6 weeks [12].

## 4. Conclusion

Neurologic manifestations of brucellosis are unusual, and sometimes diagnosis is delayed due to the unspecific symptomatology which mimics a wide array of other pathologies. It is important to take in consideration serologic *Brucella* sp. tests, including their seroconversion, in the diagnostic strategy due to the challenging diagnosis.

Finally, neurobrucellosis should be a differential diagnosis that any clinician must consider in patients coming from endemic areas with an unspecific neurologic or systemic clinical manifestation, with a granulomatous lesion not explained by other causes and with serology compatible for *Brucella* sp. infection.

## Data Availability

No date were used to support this study.
